# Croatian Translation and Validation of the Patient Satisfaction with Nursing Care Quality Questionnaire (PSNCQQ)

**DOI:** 10.3390/healthcare12090888

**Published:** 2024-04-25

**Authors:** Marin Mamić, Hrvoje Vidić, Tihomir Jovanović, Slavka Galić, Ivana Jelinčić, Štefica Mikšić, Božica Lovrić, Ivanka Zirdum, Kristijan Matković, Goran Zukanović, Goranka Radmilović, Zrinka Puharić, Mirela Frančina, Robert Lovrić, Ivan Vukoja

**Affiliations:** 1 General County Hospital Požega, Osječka 107, 34 000 Požega, Croatia; hrvoje.vidic@gmail.com (H.V.); bozica.lovric@pozeska-bolnica.hr (B.L.); ivanka.zirdum@pozeska-bolnica.hr (I.Z.); kristijan.matkovic@pozeska-bolnica.hr (K.M.); goran.zukanovic@pozeska-bolnica.hr (G.Z.); goranka.radmilovic@pozeska-bolnica.hr (G.R.); mirela.francinamf@gmail.com (M.F.); 2 Faculty of Medicine, Josip Juraj Strossmayer University of Osijek, J. Huttlera 4, 31 000 Osijek, Croatia; tihomir.jovanovic@ozbpakrac-bhv.hr (T.J.); ivana.jelincic@kbco.hr (I.J.); 3 Faculty of Dental Medicine and Health Osijek, Josip Juraj Strossmayer University of Osijek, Crkvena 21, 31 000 Osijek, Croatia; smiksic@fdmz.hr (Š.M.); zpuharic@vub.hr (Z.P.); rlovric@fdmz.hr (R.L.); 4 General Hospital Pakrac and Hospital of Croatian Veterans, Bolnička 74, 34 550 Pakrac, Croatia; 5 The Department of Social Sciences and Humanities, University of Slavonski Brod, 35000 Slavonski Brod, Croatia; slavka.galic@pozeska-bolnica.hr; 6 Department of Integrative Psychiatry, University Hospital Centre Osijek, J. Huttlera 4, 31 000 Osijek, Croatia; 7 Faculty of Medicine, University of Rijeka, Ul. Braće Branchetta 20/1, 51 000 Rijeka, Croatia; 8 Department of Nursing, University of Applied Sciences Bjelovar, 43 000 Bjelovar, Croatia

**Keywords:** nursing care, patient satisfaction, reliability and validity

## Abstract

Patient satisfaction is a crucial measure of the quality of healthcare, especially with regard to nursing services in hospital settings. Understanding and increasing patient satisfaction with nursing care is critical to improving overall healthcare and ensuring positive patient experiences during their hospital stay. The aim of this research was to evaluate the psychometric properties of the Croatian version of the Satisfaction with Nursing Care Quality Questionnaire (PSNCQQ), test the reliability and validity of the tool after translation, and investigate differences in patient satisfaction based on demographic variables, as well as their contribution to satisfaction with the quality of nursing care. After translation and adaptation, the Croatian version of the PSNCQQ was applied to 350 hospitalized patients (average age 51.19 years (range: 18–87)), of whom 194 (55.4%) were men and 156 (44.6%) were women. The results showed high internal consistency (Cronbach’s α = 0.977) and confirmed the one-factor structure of the questionnaire, explaining 70.64% of the total variance. Confirmatory factor analysis supported the unidimensional model, showing strong fit indices (χ^2^ = 583.047, df = 149, *p* < 0.001, GFI = 0.861, AGFI = 0.818, NFI = 0.936, TLI = 0.946, CFI = 0.955, RMSEA = 0.080, PCLOSE < 0.001). In conclusion, the validation of the PSNCQQ in the Croatian language increases resources for evaluating and improving the quality of nursing care. This research lays the foundation for future studies and practical applications aimed at improving patient satisfaction and nursing care outcomes in Croatia, but there are also limitations to this study, including its one-institution scope, the possible influence of factors outside the current treatment on satisfaction, and the lack of comparison with objective clinical indicators.

## 1. Introduction

Quality of healthcare is a multidimensional issue that encompasses various characteristics dependent on the performance of provided services as well as the subjective assessment of the recipients of those services. Clarifying the concept of quality is crucial to facilitating the understanding of and improvement in healthcare quality. The concept of quality healthcare has been a subject of debate within the scientific community for many years. It encompasses various dimensions of healthcare, including interpersonal and technical aspects of care, patient outcomes, structural elements, processes, and quality standards [1,2,3,4]. Additionally, indicators of high-quality care in clinical settings include patient satisfaction, safety, patient-centered care access, staff competency, and patient involvement in decision-making processes [5].

Patient satisfaction represents a key measure of healthcare quality in hospital settings, especially concerning medical nursing services. Patient satisfaction with the medical care provided is of significant importance, as it is a crucial factor influencing overall patient satisfaction during their hospital stay [6,7]. Research shows that patient satisfaction with nursing care is the most important predictor of overall patient satisfaction during their healthcare experience [8,9,10]. The reasons for this lie in the fact that nurses and technicians spend most of their time with patients in inpatient healthcare facilities, which makes them one of the most important factors in the healthcare system [10].

The quality of nursing care includes physical access to patients, meeting their psychosocial needs, satisfaction with specific attention, and the comprehensive and multidimensional responsibility of nurses to the assigned care and patient trust [11]. The quality of nursing care can be defined as the response of nurses to the physical, psychological, emotional, social, and spiritual needs of patients in order to enable them to return to a healthy and normal state [12,13]. On the other hand, patient satisfaction with the quality of nursing care is a subjective perception of care compared to expectations. During hospitalization, patient satisfaction represents a balance between the patient’s perception and their care expectations [14,15,16]. Although subjective patient perception is a concrete criterion for evaluating the quality of nursing care, many factors can influence the patient’s assessment of quality, such as personal characteristics including cultural background, level of social support, and demographic factors such as age, gender, and education [17,18].

Patient satisfaction with the quality of nursing care reflects the relationship between the perceived care they received and the level of care patients expect upon arrival at the health facility [4]. It involves a balance between expectation and perception. In other words, patient satisfaction can be considered a subjective assessment of the patient’s cognitive and emotional response resulting from the interaction between expectations and perceptions of actual care [10].

Patient satisfaction surveys are valuable tools for identifying areas where care and healthcare services can be improved. They serve as a means of understanding patient expectations, gathering suggestions, and receiving feedback, thereby contributing to the improvement in overall healthcare delivery [19,20,21].

For the same reason, attempts have been made to create questionnaires that could measure the quality of care while attempting to cover all aspects of the care provided. The first such attempt was made by Nancy Risser in 1975 [22]. For a long time, no instrument in this area had undergone psychometric testing, often due to a lack of understanding of the concept itself and the theory of patient satisfaction with nursing care [23]. The most commonly used instruments for measuring patient satisfaction with nursing care quality today include the Newcastle Satisfaction with Nursing Scale (56 items), the La Monica–Oberst Patient Satisfaction Scale (42 items), and the Patient Satisfaction with Nursing Care Quality Questionnaire (PSNCQQ) [10,24,25]. All of these tools have been widely applied and validated across various countries [10].

Since 2003, healthcare institutions in the Republic of Croatia with over 40 employees have operated nursing care as a public service, subject to compliance with education and quality standards [25,26]. Patient satisfaction has emerged as a top priority in healthcare systems and facilities worldwide, serving as a crucial indicator of the quality of the healthcare services provided [26].

But in the Republic of Croatia, there are no validated questionnaires that measure the quality of nursing care, and the need for such a questionnaire is very high. Incorporating a validated questionnaire for assessing nursing care quality in Croatia would not only address a critical gap in nursing care assessment tools but also enhance the overall quality of care delivery. By systematically measuring patient satisfaction with nursing care, healthcare facilities can identify areas for improvement and implement targeted interventions to enhance patient experiences and outcomes [27]. Moreover, researchers would benefit from a reliable instrument like the PSNCQQ questionnaire, enabling them to conduct rigorous studies on nursing care quality and contribute to evidence-based practice in nursing care [22]. Therefore, the development and utilization of a validated tool like the PSNCQQ questionnaire have the potential to significantly impact nursing care quality, overall healthcare quality, and patient outcomes in Croatia.

### 1.1. Literature Review

The Patient Satisfaction with Nursing Care Quality Questionnaire (PSNCQQ) has been validated in several countries [8,10,23,28,29] and has been widely used in research conducted in various hospital departments, proving to be a quality instrument for measuring this complex construct [19,30,31,32,33,34,35]. Validations of the questionnaire in all countries indicated a one-factor structure, except for the Arabic version, which suggested the existence of two factors: “satisfaction with the care provided” and “satisfaction with the information provided” [29]. However, two items were removed from the same questionnaire: 18. Discharge instructions and 19. Coordination of post-discharge care. As mentioned earlier, other checks indicated the presence of a single factor and demonstrated the excellent reliability of the questionnaire, with internal consistency coefficients ranging from 0.93 to 0.97 [8,10,23,28,29]. Factor analyses showed that the percentage of explained variance ranges from 51.7% in the Serbian version of the questionnaire and 60.3% in the Polish version to even 75–89% in the original version of the questionnaire [8,10,23,28,29]. Predictive validity revealed significant effects of the PSNCQQ on all three assessments of health services. In the Polish version of the PSNCQQ, it explained 67% of the variance in the assessments of the overall quality of nursing care and services, while in the original version it was 64%; in the Polish version, 57% of the variance in assessments of the overall quality of healthcare in the department was explained, while in the original version it was 73.1%; and 57% of the variance in the willingness to recommend the hospital to others was explained, while in the original version it was 55.2% of the variance [8,10].

The results of research using the PSNCQQ questionnaire on the possible connection between demographic variables and satisfaction with the quality of nursing care are contradictory. It was found that the age and level of education of the respondents can affect satisfaction with the quality of nursing care for patients, whereby satisfaction increases with age, while respondents with a higher level of education are 78% less satisfied with the quality of care compared to those with a lower level of education [23]. However, there are conflicting results showing that more educated respondents are more satisfied with the quality of care [19,32]. There are also studies conducted with the PSNCQQ questionnaire that showed that male patients are satisfied with the quality of nursing care [17], while the rest showed no gender differences [31].

Therefore, the research results are inconsistent and indicate the need for further research on satisfaction with the quality of nursing care in order to determine all the factors that may contribute to this important construct.

### 1.2. Aims

The objectives of this study were the following:

Assessing the psychometric properties of the Croatian version of the Patient Satisfaction Nursing Care Quality Questionnaire (PSNCQQ).

Evaluating the satisfaction with the quality of nursing care and checking the reliability and validity of the tool after translation.

Investigating patients’ satisfaction with the nursing care they received and examining the differences between patient satisfaction and various patient characteristics such as gender, age, education level, and place of residence.

Investigating the contribution of demographic variables (gender, age, education level, place of residence, and perceived health status) to satisfaction with the quality of nursing care.

## 2. Methods

### 2.1. Croatian Translation and Cultural Adaptation of the PSNCQQ

Before adapting the questionnaire, the author of the PSNCQQ questionnaire was requested to provide formal written consent, which we subsequently received, for its use and validation in the Croatian context. Recognizing the extreme importance of preserving the validity and reliability of the results, special emphasis was placed on ensuring that both the language and content of the measurement instrument corresponded to the cultural nuances and idiosyncrasies of the target population. Therefore, during the process of translating the PSNCQQ into the Croatian language, cultural peculiarities and specificities were carefully taken into account, guided by the comprehensive framework outlined by Wild et al. [36].

To achieve this, a rigorous translation procedure was implemented [37]. Initially, two independent translations of the original questionnaire into Croatian were carried out by experienced English language teachers, each offering a unique perspective. A later harmonized version was synthesized from these initial translations, trying to capture the essence of the original while taking cultural nuances into account. This amalgamated version was then back-translated into English to ensure linguistic fidelity and coherence. Any differences between the original and translated versions were carefully examined and reconciled to maintain consistency and accuracy.

Further refinement of the questionnaire involved seeking feedback from professionals directly involved in the provision of nursing care [36,37]. The penultimate version of the questionnaire was distributed to a selected group of 15 nurses employed at the Požega General Hospital, who were invited to review it and provide their insights. Their invaluable feedback, which included suggestions for clarity, relevance, and cultural appropriateness, was carefully considered in subsequent revisions.

Finally, the refined version of the questionnaire was validated through testing with the target population. A group of 30 patients was selected to evaluate the clarity and comprehensibility of the questionnaire items. Their feedback was crucial in confirming the appropriateness and effectiveness of the final version for the intended cultural context, ensuring that the adapted PSNCQQ accurately captured the perspectives and experiences of Croatian healthcare users.

### 2.2. Study Design

A cross-sectional study was conducted. The research was conducted at the Požega General Hospital from 2022 to 2023. The questionnaires were distributed to the participants upon discharge from the hospital. Each participant had to sign their voluntary consent to participate in the research. The inclusion criteria were age over 18 years, the ability to independently understand and complete the questionnaire, and consent to participate in this study, while the exclusion criteria were age under 18 years, inability to independently understand and fulfill the questionnaire, and refusal to participate in this study. 

### 2.3. Participants

A total of 374 participants took part, of whom 350 correctly completed the questionnaire and were included in this study. Since the PSNCQQ consisted of 19 items, the final sample size was deemed adequate according to the response criteria, with eighteen respondents for each of the items.

### 2.4. Instruments

The patient demographics questionnaire includes data on age, gender, marital status, level of education, place of residence, and self-assessment health at admission (on a 5-point Likert scale ranging from 1—“Very poor” to 5—“Excellent”). The questionnaire was created for research purposes.

The PSNCQQ (Patient Satisfaction with Nursing Care Quality Questionnaire) consists of three parts and measures patient satisfaction with the quality of nursing care [10]. The first part of the questionnaire consists of 19 items related to the assessment of the quality of nursing care. Participants respond by assessing their level of agreement with each statement on a Likert-type scale from 1—“Excellent” to 5—“Poor” [10]. The questions are recoded and the first part of the questionnaire is calculated as the sum of all responses, where a higher score indicates a higher assessment of the quality of nursing care by the participants

The second part of the questionnaire consists of four questions related to the assessment of the overall quality of service received during hospitalization, the assessment of the overall quality of nursing care received during hospitalization, the assessment of their health condition upon admission to the hospital, and whether they would recommend the nursing care facility to their friends and family for further treatment. Participants respond to questions about the overall quality of service, quality of nursing care, and health condition by assessing their level of agreement with each statement on a Likert scale ranging from 1—“Excellent” to 5—“Poor”. For the question about recommending the healthcare facility, participants respond by assessing their level of agreement with each statement on a Likert scale ranging from 1—“Strongly agree” to 5—“Strongly disagree”. The third part of the questionnaire relates to demographic questions, which are already included in the demographic questionnaire and will not be used in this study [10].

The third part of the questionnaire addresses the length of hospitalization, mode of admission (emergency, regular), number of hospitalizations in the hospital in the previous two years, and number of persons in the room during treatment [10].

### 2.5. Statistical Methods

Descriptive statistical methods were used to describe the frequency distribution of the examined variables. Mean values are expressed as arithmetic mean, standard deviation, and range. Predictive validity was assessed by multiple regression analysis, the Mann–Whitney test, and the Kruskal–Wallis test. The Mann–Whitney test was applied to examine differences between two independent groups of participants, while the Kruskal–Wallis test investigated differences between multiple independent variables. Hierarchical regression analysis was performed to assess how well the total score of the PSNCQQ-Cro questionnaire predicts the overall quality of nursing care, the assessment of the quality of medical care measured by a single item, and the likelihood of recommending a health facility to acquaintances and friends. Furthermore, linear regression analysis was used to determine predictors of satisfaction with nursing care quality.

The reliability of the questionnaire was analyzed using Cronbach’s coefficient, confirming the internal consistency by examining the mean correlations of each item (inter-item) and between the items and the total score (item–total). Bartlett’s test of sphericity and the Kaiser–Meyer–Olkin (KMO) measure of sampling adequacy were used to assess the factorability of the data. The construct validity of the questionnaire was confirmed by exploratory factor analysis, whereby confirmatory factors were used to test how well the measured variables represented the number of constructs. To test the normality of the distribution, the Kolmogorov–Smirnov test was used, with the significance level set at *p* < 0.05. SPSS v.25 and AMOS v.21 were used for data analysis.

## 3. Results

The average age of the participants was 51.19 years (range from 18 to 87 years; SD = 17.043), with 194 (55.4%) men and 156 (44.6%) women, of whom 208 (59.4%) had a secondary level of education and 181 (51.7%) lived in urban areas. As for previous treatments, 139 (39.7%) were admitted via emergency hospital admission, while 198 (56.6%) were previously planned treatments. During hospitalization, 61 (17.4%) participants were alone in a room, while 133 (38%) shared a room with one other patient, and 156 (44.6%) shared a room with two other patients. Furthermore, 198 (56.6%) were treated for the first time in this institution, while 88 (24.1%) were treated once, and 35 (10%) more than once, including the current stay. The average length of stay was 6.926 days (range 1–31; SD = 4.887).

Participants expressed the quality of nursing care using a set of 19 items. The highest level of agreement was found for item no. 14 “Skills and competence of nurses” (M = 4.248, SD = 0.953), while the lowest level of agreement was found for item no. 6 “Involvement of family in care”(M = 3.765, SD = 1.036) (Table 1).

### 3.1. Reliability Analysis

The coefficients of internal consistency for the entire questionnaire indicated high reliability of the overall score across all items (α = 0.977). Additionally, the average inter-item correlation was found to be 0.688, suggesting a high degree of consistency among the questionnaire items. These results further support the reliability of the instrument and its ability to measure the intended construct effectively (Table 2).

### 3.2. Factor Analysis

Before proceeding with further analysis, it was essential to confirm that all prerequisites for conducting factor analysis were met. The Kaiser–Meyer–Olkin (KMO) measure, which assesses the sampling adequacy for factor analysis, yielded a highly satisfactory value of KMO = 0.974. This surpasses the recommended threshold of 0.5, indicating that the data are highly suitable for factor analysis. Additionally, Bartlett’s test of sphericity was significant (χ^2^ (df = 171) = 7197.761; *p* < 0.001), suggesting that the correlation matrix significantly differs from the identity matrix. This outcome supports the presence of significant relationships among variables, further validating the appropriateness of the data for factorization. Together, these results provide strong assurance that the correlation matrix is suitable for factor analysis, ensuring the validity and reliability of the subsequent factor analysis outcomes (Table 3).

**Figure 1:**
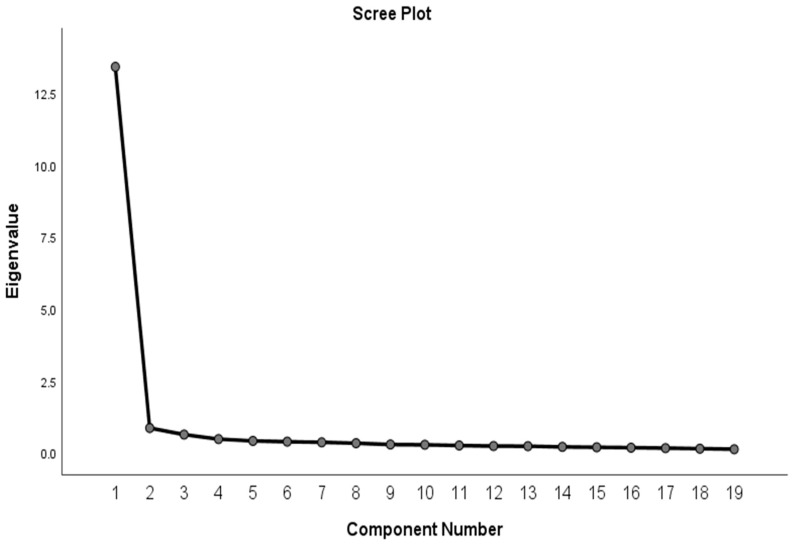
Scree plot of the components for the 19 variables of the factor analysis.

An exploratory factor analysis was conducted to identify common factors. The analysis revealed that the single-factor questionnaire explained 70.64% of the shared variance. Regarding individual-item factor loadings, item 6 exhibited a loading of 0.469, while other items ranged from 0.591 to 0.778. These findings indicate that all items had satisfactory factor loadings, exceeding the threshold of 0.30 (Figure 1 and Table 4).

### 3.3. Confirmatory Analysis

**Figure 2:**
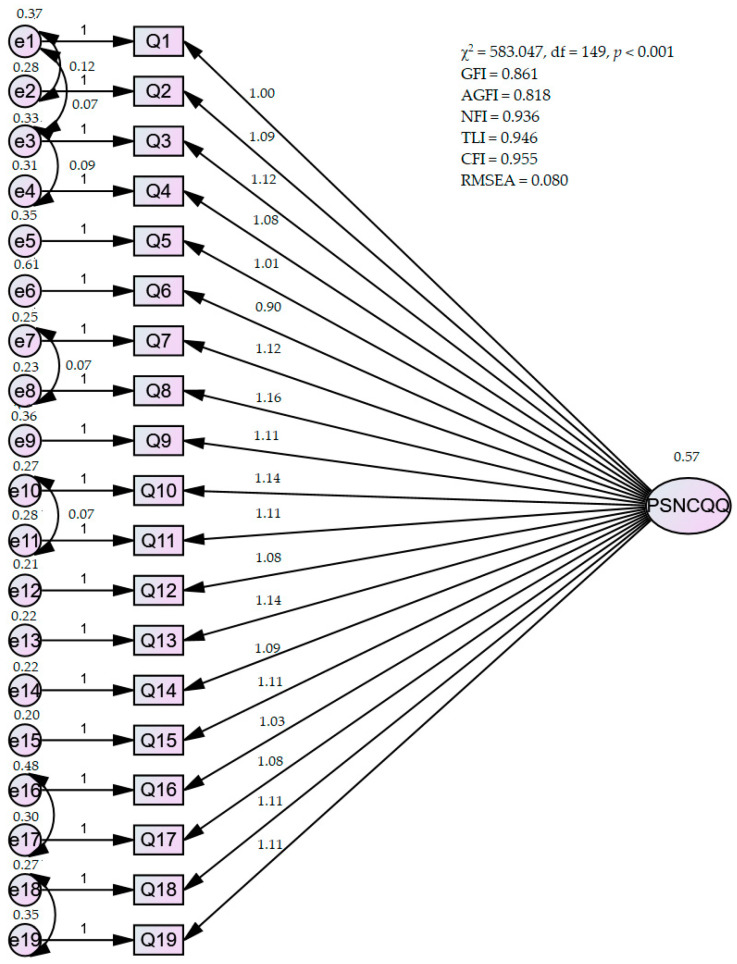
Confirmatory factor analysis of PSNCQQ.

To determine the factor structure, a single-factor multilevel CFA was conducted with all 19 items sharing one common factor. Additionally, correlating errors for similarly worded test items were explored to enhance model fit. The initial single-factor model yielded a very poor fit, as evidenced by χ^2^ = 726.949, df = 152, *p* < 0.001, GFI = 0.628, AGFI = 0.732, NFI = 0.901, TLI = 0.910, CFI = 0.920, and RMSEA = 0.104, with PCLOSE < 0.001. However, after correlating errors for the similarly worded test items, the single-factor model showed improvements in fit indices, with χ^2^ = 583.047, df = 149, *p* < 0.001, GFI = 0.861, AGFI = 0.818, NFI = 0.936, TLI = 0.946, CFI = 0.955, and RMSEA = 0.080, with PCLOSE < 0.001 (Figure 2).

### 3.4. Predictive Validity

The predictive validity of the PSNCQQ-Cro was assessed through its impact on three additional patient evaluations of healthcare services. Separate multiple regression models were conducted, with the PSNCQQ-Cro and potentially significant intervening variables such as age, gender, length of stay, and self-reported health status upon admission to the hospital included as predictor variables. The dependent variables were the overall quality of care and services, the overall quality of nursing care in the ward, and the willingness to recommend the hospital to family and friends. After controlling for length of stay, gender, age, and self-rated health, the PSNCQQ-Cro accounted for substantial proportions of the variance in the overall quality of care and services (54.3%), the overall quality of nursing care (54.2%), and the intention to recommend the hospital to family and friends (42.8%) across the combined hospital sample (Table 5).

The results indicated a significant difference in satisfaction with nursing care quality based on participants’ gender (Mann–Whitney test; *p* = 0.049), with men reporting significantly higher satisfaction compared to women. Significant differences were also observed based on participants’ level of education (Kruskal–Wallis test; *p* = 0.002). Post hoc comparisons (Dunn) revealed significantly lower satisfaction among participants with elementary education compared to those with secondary vocational education (*p* = 0.044) and higher education (*p* = 0.013). Furthermore, there was a significant difference in satisfaction with nursing care quality based on participants’ age (Kruskal–Wallis test; *p* < 0.001). Participants aged 71 and older reported significantly lower satisfaction compared to those aged 61 to 70 (*p* = 0.010), 51 to 60 (*p* < 0.001), 41 to 50 (*p* < 0.001), 31 to 40 (*p* < 0.001), and 18 to 30 (*p* < 0.001) (Table 6).

To determine the predictors of nursing care quality from demographic variables (gender, age, place of residence, level of education, and health status), linear regression analysis was used. The results showed that the included variables significantly explained 14.9% (adjusted R-squared = 0.149; *p* < 0.001) of the variance in satisfaction with nursing care quality. The age of the respondents (*p* = 0.001) and their health-at-admission status (*p* < 0.001) were found to be significant predictors. Upon examination of the β coefficient, it is evident that age contributes negatively, while the assessment of health status contributes positively to satisfaction with nursing care quality (Table 7).

## 4. Discussion

The aim of this research was to assess the psychometric properties of the Croatian version of the Patient Satisfaction with Nursing Care Quality Questionnaire (PSNCQQ-Cro) in order to assess the reliability and validity of this instrument in measuring patient satisfaction with the quality of nursing care in a sample of patients in Croatia. The aim was also to provide a detailed analysis of internal consistency, factor structure, and correlation between individual items to ensure that the Croatian version of the PSNCQQ meets the standards of reliability and validity required for use in research and clinical settings in Croatia.

The results showed that the Croatian version of the PSNCQQ has satisfactory psychometric properties, in accordance with the findings of studies conducted on the original questionnaire and its validations in other countries. The Cronbach’s α coefficient of internal consistency for all 19 items was 0.977, and the correlations between individual items and the total score ranged from 0.657 to 0.866. These results are comparable to the values of the Cronbach’s α coefficient of the original questionnaire (α = 0.97) [10] and validation in other countries, which ranged from 0.94 to 0.97, while the correlations between individual items and the total score ranged from 0.56 to 0.89 [8,23,28,29]. However, it is important to interpret these results with caution. While high Cronbach’s α values suggest strong internal consistency, there is a possibility that certain items may be redundant rather than truly measuring the same construct. Further investigations of the underlying structure of the scale to be provided later, such as exploratory factor analysis, could provide insight into item redundancy and inform possible modifications to improve scale performance [38].

In our exploratory factor analysis (EFA), we observed a range of factor loadings across the items included in our questionnaire, reflecting their association with the underlying latent constructs. It is important to note that item 6 showed the lowest factor loading of all items, with a loading of 0.469, while the other items showed a minimum loading ranging from 0.591 to 0.778. This discrepancy in factor loadings calls into question the contribution of item 6 to the measurement of the latent construct under investigation. But item 6, despite its lower factor loading, can still provide valuable information about the construct. However, its relatively weaker association with the underlying factor suggests that it may not be as strong an indicator of the construct as other items in the scale. One of the possible explanations for the lower factor loading of item 6 could be its lower mean compared to other items. The mean item score reflects the average response of all respondents, and a lower mean score suggests that respondents gave lower scores on average for that particular item. This may mean that respondents perceive item 6 differently or that it is less representative of the construct compared to other items [38].

Factor analysis confirmed the one-factor structure of the questionnaire, explaining a significant percentage of the total variance. These findings are in accordance with the validation of the original PSNCQQ questionnaire and the validations in other countries, further confirming the validity and reliability of the Croatian version of this instrument [10,23,28]. However, it is important to note that these results differ from the findings of the validation of the Arabic version, which suggested the presence of two factors [29]. This difference in results may indicate cultural or linguistic differences in the understanding and interpretation of individual questionnaire items among different populations.

In order to assess model fit in confirmatory factor analysis (CFA), various model fit indices were evaluated. It is important to note that correlated error terms are included in the CFA model to account for method effects or shared method variance among items that tap similar constructs. Correlated error patterns among indicators are often encountered in practical scenarios. If there is a legitimate reason for correlation between indicator error terms, this can be addressed in a structural equation model [39]. Potential factors influencing responses include silent response bias, assessment methods such as questionnaires or observer ratings, the presence of reversed or similarly worded test items or tapping closely related constructs, and individual characteristics such as reading difficulties or cognitive biases such as groupthink, all of which can affect the ability of respondents to give truthful answers to the questionnaire [38,39]. In our case, when test items are similarly worded, respondents may have difficulty distinguishing them, leading to ambiguous responses and measurement errors. This ambiguity can introduce systematic biases into the data, affecting the accuracy of the measuring instrument. Furthermore, if similarly worded items measure different aspects of the construct, their inclusion may inflate the apparent reliability of the instrument while compromising its validity [38,39].

As a baseline test, the one-factor multilevel CFA provided a very poor fit, but after correlating the errors for similarly worded test items, they showed that the one-factor model provided an adequate fit. According to recommended guidelines, RMSEA values below 0.05 are considered excellent, values between 0.05 and 0.08 are acceptable, values between 0.08 and 0.1 are marginal, and values greater than 0.1 are considered poor [40]. The observed RMSEA value of 0.080 in this data set suggests an acceptable fit. Although the GFI value for this data set is 0.86, which is below the threshold of 0.9, it is important to note that the GFI and AGFI values can vary depending on the sample size. A CFI value of 0.95 indicates a good fit. Although it is recommended that the NFI and TLI indices be above 0.9 for a good fit, both values meet this criterion. Overall, based on these indices, the fit of the one-factor model to this data set is considered acceptable [41].

This CFA suggests that the factor structure fits the original questionnaire model and is consistent with the original questionnaire and its validations in other cultures. This result further confirms the questionnaire and indicates its reliability and applicability in the Croatian context.

The predictive validity of the PSNCQQ-Cro was assessed by assessing its ability to predict expected outcomes commonly used for validation in health services research. The results related to the predictive validity of the overall quality of care and services, the overall quality of healthcare in the department, and the willingness to recommend the hospital to family and friends showed slightly lower values compared to the validation of the original version of the questionnaire [10] and the validation of the questionnaire in other countries [8]. These results provide strong support for the predictive validity of the PSNCQQ-Croatian. Possible causes of slightly lower values of predictive validity may be related to different characteristics of the respondents or the context in which this research was conducted. For example, variations in demographic characteristics, such as age, gender, or the socioeconomic status of respondents, may influence their perception of nursing care quality [17,18,23].

As for the other findings of this research, it was observed that female subjects expressed significantly lower satisfaction with the quality of nursing care compared to men, which is in line with the validation of the Serbian version of the PSNCQQ [23] as well as with other studies on the quality of nursing care [10,42,43]. The explanation for the lower satisfaction rate among female patients may lie in social norms and expectations regarding gender roles. Women are often seen as caregivers and are expected to prioritize hygiene and healthcare, which could influence their perception and evaluation of health experiences. In addition, women may spend more time in bed and show more passivity in medical facilities, factors that may affect their level of satisfaction compared to men [10].

It was also determined that respondents with a lower level of education expressed lower satisfaction compared to respondents with a higher level of education. These results contradict the validation of the Serbian version of the questionnaire [23]. In addition, the results of previous studies on this topic are inconsistent; some studies show that patients with a lower level of education are more satisfied with the quality of nursing care [23], while others suggest the opposite, with more educated respondents showing greater satisfaction with the quality of nursing care or the quality of healthcare [44,45]. This could be due to the fact that individuals with a higher level of education have a greater awareness of the services offered by the hospital compared to those with a lower level of education [46]. The existing literature suggests that higher education fosters greater sensitivity to the care and services provided by the healthcare system, potentially leading to higher satisfaction scores [45]. Some research has also suggested that poorer health literacy may affect satisfaction with nursing care [47], and as research has shown that people with a lower level of education have poorer health literacy, this could be one of the reasons for these results [48]. Despite everything, the inconsistency in the results points to the need for further research into this relationship.

There is also a difference in satisfaction based on the age of the respondent; repeat respondents aged 71 years and older showed worse assessments of the quality of nursing care compared to respondents of all other younger ages. It was also found that age has a negative effect on satisfaction with the quality of nursing care. The results of other studies on this topic are inconsistent; while some studies show that younger respondents are more satisfied [44], others suggest the opposite, whereby older respondents are more satisfied with the quality of nursing care, as in the Serbian version of the questionnaire [23]. Possible reasons for these results could be that, as individuals age, their need for medical care and overall healthcare increases. More frequent visits to medical institutions and greater demands on healthcare providers can lead to greater dissatisfaction if their expectations are not met. As patients continue to age and develop more health problems that require more healthcare services and place greater demands on providers, their satisfaction may decline. However, it remains unclear whether age-related variations in satisfaction result from differences in patient expectations, perceptions of care, or actual differences in quality of care. Further research on this issue is needed to elucidate the mechanisms driving these associations. By conducting additional research, we can gain a deeper understanding of how age affects patient satisfaction with nursing care, thereby informing the development of targeted interventions to address the specific needs of different patient demographics.

It was also determined that the state of health at admission contributes positively to satisfaction with the quality of nursing care. The results are consistent with other studies that suggest a similar relationship, but this relates to the quality of healthcare rather than the quality of nursing care. However, since it is a self-assessment of the state of health, the question remains whether the patients are really in a bad state of health or if it is just their subjective feeling. Non-health-related factors could potentially influence satisfaction, as both rely on patient self-reports. For example, individuals with a generally pessimistic outlook on life may tend to perceive their health negatively and express dissatisfaction with their nursing care services [49]. It is also possible that patients with poorer health status may be more critical of the medical care they receive, leading to lower levels of satisfaction, as they may have higher expectations or require more intensive care [50].

The current study offers valuable insights into patient satisfaction within a specific timeframe. However, longitudinal research could improve our understanding by tracking fluctuations in satisfaction levels over time. This longitudinal approach would provide a more comprehensive view of how health interventions and changes in nursing care practice affect patient perceptions of quality. By observing trends and patterns in patient satisfaction over an extended period, healthcare providers can identify areas for improvement and adjust interventions accordingly. Furthermore, longitudinal research enables the long-term effectiveness and sustainability of implemented changes to be assessed, providing valuable guidance for ongoing quality improvement efforts in healthcare facilities.

## 5. Conclusions

In conclusion, on the basis of the aforementioned, the validation of the PSNCQQ in the Croatian language leads to new tools for assessing and improving the quality of nursing care. The results of this survey are a good foundation for further research as well as an opportunity to develop strategies within healthcare systems that may help improve patients’ satisfaction with treatment and enhance healthcare services. Moreover, this study facilitates interventions targeted at evidence-based practices that could be designed to address individual patient needs and preferences, thus offering health policy makers and health professionals guidance on how to come up with strategies that will lead to better patient satisfaction with treatment outcomes as well as enhanced quality of care.

This research will give insight into the reliability and suitability of the PSNCQQ-Cro questionnaire for use in Croatian healthcare. Rigorous reliability analyses and factorial and confirmatory analyses were performed, and the questionnaire demonstrated strong validity in measuring satisfaction with nursing care quality. However, it is also important to highlight that the ease of use and adaptability of this questionnaire to different environments make it a good tool for monitoring patient satisfaction among healthcare institutions, while its brevity reduces the burden on respondents during the survey, thus enabling wider use. These findings may influence areas for development within the delivery of medical care and empower hospitals and healthcare facilities to tailor interventions to meet the individual needs of each patient. The PSNCQQ-Cro questionnaire appears to be an invaluable instrument that aims at improving treatment practices and outcomes, given the significant role played by hospital treatment giants in healthcare systems.

This study allows ongoing monitoring of patient satisfaction trends, identification of areas for improvement, and implementation of interventions that will lead to better nursing care delivery and patient outcomes across the country.

## 6. Limitations to this Study

Although the established validity and reliability indicators of the PSNCQQ-Cro suggest good metric characteristics and utility for future research, it should be mentioned that there are certain limitations to this study. This research was conducted at only one health institution, which may limit the generalizability of the findings. In the future, it would be good to try to repeat this research in several different health institutions in Croatia in order to ensure the validity and reliability of the questionnaire in different contexts. In addition, longitudinal studies could provide important insights into the stability and consistency of questionnaire outcomes over time, offering a deeper understanding of the lasting effects of interventions aimed at improving nursing care quality and patient satisfaction. Additionally, it would be good for future research to further assess the reliability of the questionnaire using the test–retest method along with the internal consistency method. This would give insight into the stability of the perception of the quality of nursing care over a certain period of time.

Also, one of the limitations of this study is that factors unrelated to current treatment can potentially affect satisfaction with the quality of nursing care. In order to avoid such potential limitations, further research in this area is important, in which various factors that could affect patient satisfaction with the quality of nursing care would be investigated, and thus the interrelationship of these constructs could be better understood. Also, a potential error in research could be avoided by comparing it with objective clinical indicators of care carried out by health institutions.

All the mentioned additional procedures could strengthen the validity and reliability of the questionnaire, but they would also provide valuable insights for improving the quality of healthcare in different health institutions.

## Figures and Tables

**Table 1:** Descriptive statistics of PSNCQQ questionnaire items.

	M (Range)	SD
Q1—Information you were given	3.982 (1–5)	0.978
Q2—Instructions	4.082 (1–5)	0.984
Q3—Ease of getting information	4.068 (1–5)	1.021
Q4—Information given by nurses	4.094 (1–5)	0.992
Q5—Informing family or friends	3.911 (1–5)	0.969
Q6—Involving family or friends in your care	3.765 (1–5)	1.036
Q7—Concern and caring by nurses	4.300 (1–5)	0.986
Q8—Attention of nurses to your condition	4.148 (1–5)	0.998
Q9—Recognition of your opinions	3.822 (1–5)	1.028
Q10—Consideration of your needs	4.068 (1–5)	1.010
Q11—The daily routine of the nurses	3.965 (1–5)	0.995
Q12—Helpfulness	4.214 (1–5)	0.937
Q13—Nursing staff response to your calls	4.214 (1–5)	0.982
Q14—Skills and competence of nurses	4.248 (1–5)	0.953
Q15—Coordination of care	4.228 (1–5)	0.951
Q16—Restful atmosphere provided by nurses	3.982 (1–5)	1.043
Q17—Privacy	4.068 (1–5)	0.987
Q18—Discharge instructions	4.048 (1–5)	0.990
Q19—Coordination of care after discharge	3.931 (1–5)	1.030

Note: M—mean; SD—standard deviation.

**Table 2:** Reliability coefficient and mean correlation among PSNCQQ-Cro questionnaire units.

	PSNCQQ-Cro
α	0.977
r_s_	0.688

Note: α—Cronbach’s α coefficient; r_s_—average inter-item correlations.

**Table 3:** KMO and Bartlett’s test.

KMO and Bartlett’s Test		
Kaiser–Meyer–Olkin measure of sampling adequacy	0.974	
Bartlett’s test of sphericity	Approx. chi-square	7197.761
	df	171
	*p*	<0.001

Note: KMO—Kaiser–Meyer–Olkin measure of sampling adequacy; df—Degrees of freedom; *p*—statistical significance.

**Table 4:** Factor loadings of items in the single-factor questionnaire.

Factor Loadings	Extraction
Q1—Information you were given	0.645
Q2—Instructions	0.735
Q3—Ease of getting information	0.717
Q4—Information given by nurses	0.712
Q5—Informing family or friends	0.653
Q6—Involving family or friends in your care	0.469
Q7—Concern and caring by nurses	0.755
Q8—Attention of nurses to your condition	0.778
Q9—Recognition of your opinions	0.682
Q10—Consideration of your needs	0.748
Q11—The daily routine of the nurses	0.744
Q12—Helpfulness	0.761
Q13—Nursing staff response to your calls	0.765
Q14—Skills and competence of nurses	0.749
Q15—Coordination of care	0.771
Q16—Restful atmosphere provided by nurses	0.591
Q17—Privacy	0.709
Q18—Discharge instructions	0.746
Q19—Coordination of care after discharge	0.692
Variance explained: 70.64%	

**Table 5:** Risk-adjusted final multiple regression models.

Outcome		AR^2^
Overall quality of care and services	Age, gender, LOS ^†^, health at admission	0.141 *
	PSNCQQ	0.543 *
	Final model	0.684 *
Overall quality of nursing care	Age, gender, LOS ^†^, health at admission	0.142 *
	PSNCQQ	0.542 *
	Final model	0.684 *
Intention to recommend the hospital to family and friends	Age, gender, LOS ^†^, health at admission	0.109 *
	PSNCQQ	0.428 *
	Final model	0.537 *

Note: ^†^ LOS—indicates length of stay; AR^2^—coefficient of determination; * *p* < 0.05.

**Table 6:** Differences in patient satisfaction with nursing care quality according to demographic variables.

	Satisfaction with Nursing Care Quality			
		M (Range)	SD	*p*
Gender	male	78.644 (27–95)	15.644	0.023 *
	female	75.288 (19–95)	16.195	
Age	18–30	82.415 (43–95)	12.418	<0.001 ^†^
	31–40	82.018 (46–95)	12.433	
	41–50	80.350 (39–95)	11.738	
	51–60	77.717 (19–95)	15.006	
	61–70	74.689 (28–95)	18.512	
	71 and older	64.960 (26–95)	17.643	
Education level	elementary school	71.654 (19–95)	18.680	0.007 ^†^
	vocational degree	78.081 (28–95)	14.840	
	bachelor’s degree	80.480 (35–95)	12.887	
	higher education	81.805 (29–95)	13.411	
Place of residence	urban	77.773 (27–95)	15.072	0.689 *
	rural	76.479 (19–95)	16.625	

Note: M—mean; SD—standard deviation; *p*—statistical significance; * Mann–Whitney test; ^†^ Kruskal–Wallis test.

**Table 7:** Results of linear regression analysis for satisfaction with nursing care quality as the criterion variable.

	β	t	*p*	AR^2^
(Constant)		14.111	<0.001 *	0.149 *
Gender	−0.050	−0.987	0.324	
Age	−0.190	−3.274	0.001 *	
Place of residence	−0.070	−1.392	0.165	
Educational level	0.078	1.458	0.146	

Note: β—regression coefficient; t—the size of the difference relative to the variation in the sample data; *p*—statistical significance; AR^2^—coefficient of determination; * *p* < 0.01.

## Data Availability

Data are available from the corresponding author upon reasonable request.
